# Rapid deacetylation of yeast Hsp70 mediates the cellular response to heat stress

**DOI:** 10.1038/s41598-019-52545-3

**Published:** 2019-11-07

**Authors:** Linan Xu, Nitika, Naushaba Hasin, Daragh D. Cuskelly, Donald Wolfgeher, Sean Doyle, Paul Moynagh, Sarah Perrett, Gary W. Jones, Andrew W. Truman

**Affiliations:** 1 Department of Biology, Maynooth University, Maynooth, Co. Kildare Ireland; 2 Department of Biological Sciences, University of North Carolina at Charlotte, Charlotte, USA; 3 Institute for Genome Sciences, University of Maryland Baltimore, Baltimore, USA; 4 Department of Molecular Genetics and Cell Biology, The University of Chicago, Chicago, USA; 5 National Laboratory of Biomacromolecules, CAS Center for Excellence in Biomacromolecules, Institute of Biophysics, Chinese Academy of Sciences, Beijing, 100101 China; 6 University of the Chinese Academy of Sciences, Beijing, 100049 China; 7 Centre for Biomedical Science Research, School of Clinical and Applied Sciences, Leeds Beckett University, Portland Building, City Campus, Leeds, LS1 3HE United Kingdom

**Keywords:** Chaperones, Fungal genetics

## Abstract

Hsp70 is a highly conserved molecular chaperone critical for the folding of new and denatured proteins. While traditional models state that cells respond to stress by upregulating inducible HSPs, this response is relatively slow and is limited by transcriptional and translational machinery. Recent studies have identified a number of post-translational modifications (PTMs) on Hsp70 that act to fine-tune its function. We utilized mass spectrometry to determine whether yeast Hsp70 (Ssa1) is differentially modified upon heat shock. We uncovered four lysine residues on Ssa1, K86, K185, K354 and K562 that are deacetylated in response to heat shock. Mutation of these sites cause a substantial remodeling of the Hsp70 interaction network of co-chaperone partners and client proteins while preserving essential chaperone function. Acetylation/deacetylation at these residues alter expression of other heat-shock induced chaperones as well as directly influencing Hsf1 activity. Taken together our data suggest that cells may have the ability to respond to heat stress quickly though Hsp70 deacetylation, followed by a slower, more traditional transcriptional response.

## Introduction

Cells are challenged by acute and chronic stresses such as heat shock, oxidative stress, variations in pH and salt concentrations, and other toxic chemicals. In stressed environments, proteins can unfold, aggregate or misfold, which accelerates cellular damage and can result in cell death and disease. Therefore, cells have evolved specific networks to detect, monitor and respond to such stresses from the environment^1–3^.

A rapid way to respond to cellular stress is the post-translational modifications of proteins. These modifications range in size from relatively small additions such as phosphorylation, acetylation and methylation to addition of larger protein groups such as ubiquitination and SUMOylation. Lysine acetylation is present in all organisms from bacteria to mammals, suggesting that its functions may have been maintained throughout evolutionary history^4,5^. Functionally, histone acetylation has been extensively studied in the regulation of gene expression^6,7^. Moreover, acetylation and deacetylation also play important roles in regulating cellular metabolism^8,9^, protein folding^10^, and sister chromatid cohesion^10^. Based on the evolutionarily conserved roles of acetylation, *S*. *cerevisiae* has been broadly used to study the molecular mechanisms and cellular processes that are influenced by acetylation of specific proteins. Many lysine acetyl-transferases and deacetylases were discovered in yeast and their orthologs were subsequently identified in higher eukaryotes^4,5,11^.

Molecular chaperones are also regulated through acetylation, with acetylation of several lysines on Hsp90 altering ATP binding and chaperone function of Hsp90^12^. Human HSF1 (Heat Shock Factor) which controls the global expression of chaperones also undergoes stress-induced acetylation negatively regulating its DNA-binding activity and overall cellular response to stress^13^.

Ssa1, a constitutively expressed yeast Hsp70 is highly modified by PTMs^14^. Although these modifications have been detected multiple times through global mass spectrometry studies, little is known on how these sites are regulated and the functional consequences of these modifications. T36 phosphorylation of Ssa1 dictates interaction with Ydj1 and the G1 cyclin Cln3, which subsequently regulates the degradation of this cyclin and progression through the cell cycle^15^. Oxidative modification of C264 and C303 abolishes the Ssa1-mediated repression of Hsf1 and activates a cascade resulting in the upregulation of stress-related genes^16^. Several studies on mammalian Hsp70 have identified sites of modification which effect affect dimerization, client blinding and protein folding^15,17–22^. In this study we demonstrate that yeast Ssa1 is deacetylated specifically at four key lysine residues in response to heat shock. Deacetylation of these residues results in functional and changes in Hsp70 that influence stress-associated phenotypes. We propose that this mechanism provides a rapid cellular response to heat shock, in tandem with the slower induction of chaperone proteins.

## Results

### Ssa1 is rapidly deacetylated in response to heat shock

**Figure 1:**
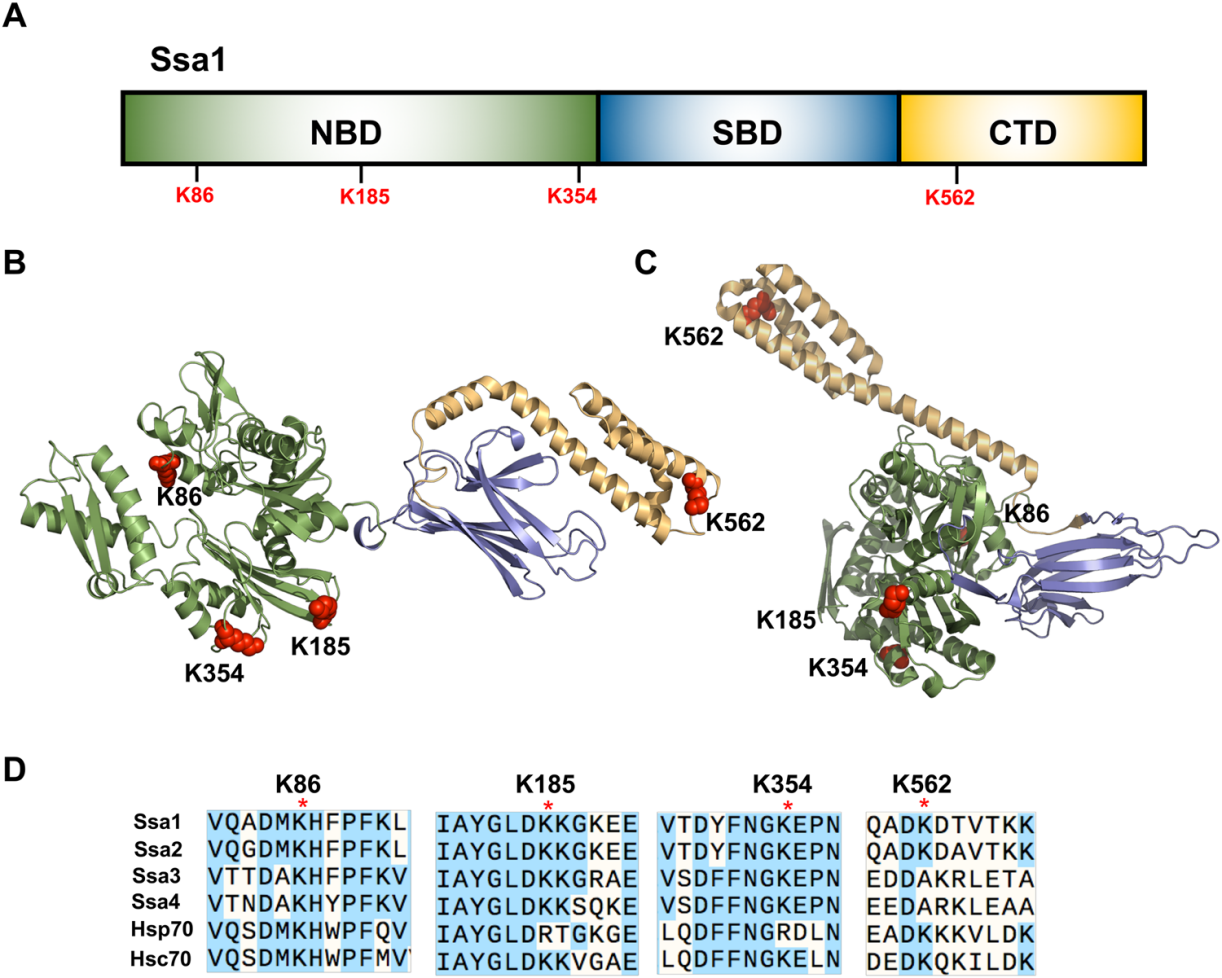
Heat shock alters acetylation of Ssa1. (**A**) Domain structure of Ssa1. All lysine residues that were found to be deacetylated upon heat shock as deretmined by mass spectrometry are indicated. (**B**,**C**) Cartoon representation of Hsp70 in the ADP-bound open conformation (PDB: 2KHO) and in the ATP-bound closed conformation (PDB: 4JNE) showing the NBD (green), SBD (blue) and CTD lid (yellow). The four deacetylated residues are highlighted in red. (**D**) Conservation of the deacetylated residues in Hsp70. amino acid sequences of *Saccharomyces cerevisiae* Hsp70 isoforms Ssa1, Ssa2, Ssa3 and Ssa4 as well as sequences for human Hsc70 and Hsp70 were aligned using Snapgene. Amino acids identified as becoming deacetylated upon heat shock are annotated with a red dot. Raw mass spectrometry data are available via ProteomeXchange with identifier PXD015185.

To investigate whether the post-translational modification (PTM) of Ssa1 differs in response to heat exposure, we analyzed Ssa1 PTMs from yeast either untreated or exposed to 37 °C for 30 mins using high-resolution quantitative mass spectrometry as^22^. Following heat shock, four residues (K86, K185, K354 and K562) were rapidly deacetylated (raw mass spectrometry data are available via ProteomeXchange with identifier PXD015185). Among them, K86 and K562 have been reported as acetylated residues, and K354 as a ubiquitinylated lysine^23^. Notably, the sites are spaced throughout the Ssa1 structure, with three of the four deacetylated residues existing in the NBD and one in the lid of SBDα (Fig. 1A–C). All four of the deacetylated lysine residues are present on the surface of Ssa1 in flexible regions of the protein with acetylation likely altering local Hsp70 structure (Fig. 1B,C). Yeast possess four closely-related cytosolic Ssa proteins that differ in expression patterns and client specificity^24^. We examined the conservation of K86, K185, K354 and K562 between the yeast Ssa proteins as well as the two major mammalian isoforms Hsp70 and Hsc70 (Fig. 1D). While K86 is maintained in all yeast and mammalian Hsp70s, there was less conservation observed for K185, K354 and K562 (Fig. 1D). K185 and K354 are replaced by arginine in mammalian Hsp70, and K562 is replaced by alanine in the inducible Ssa isoforms Ssa3 and Ssa4 (Fig. 1D). None of these amino acid substitutions are capable of undergoing acetylation.

### Deacetylation of Hsp70 enhances thermotolerance in yeast

**Figure 2:**
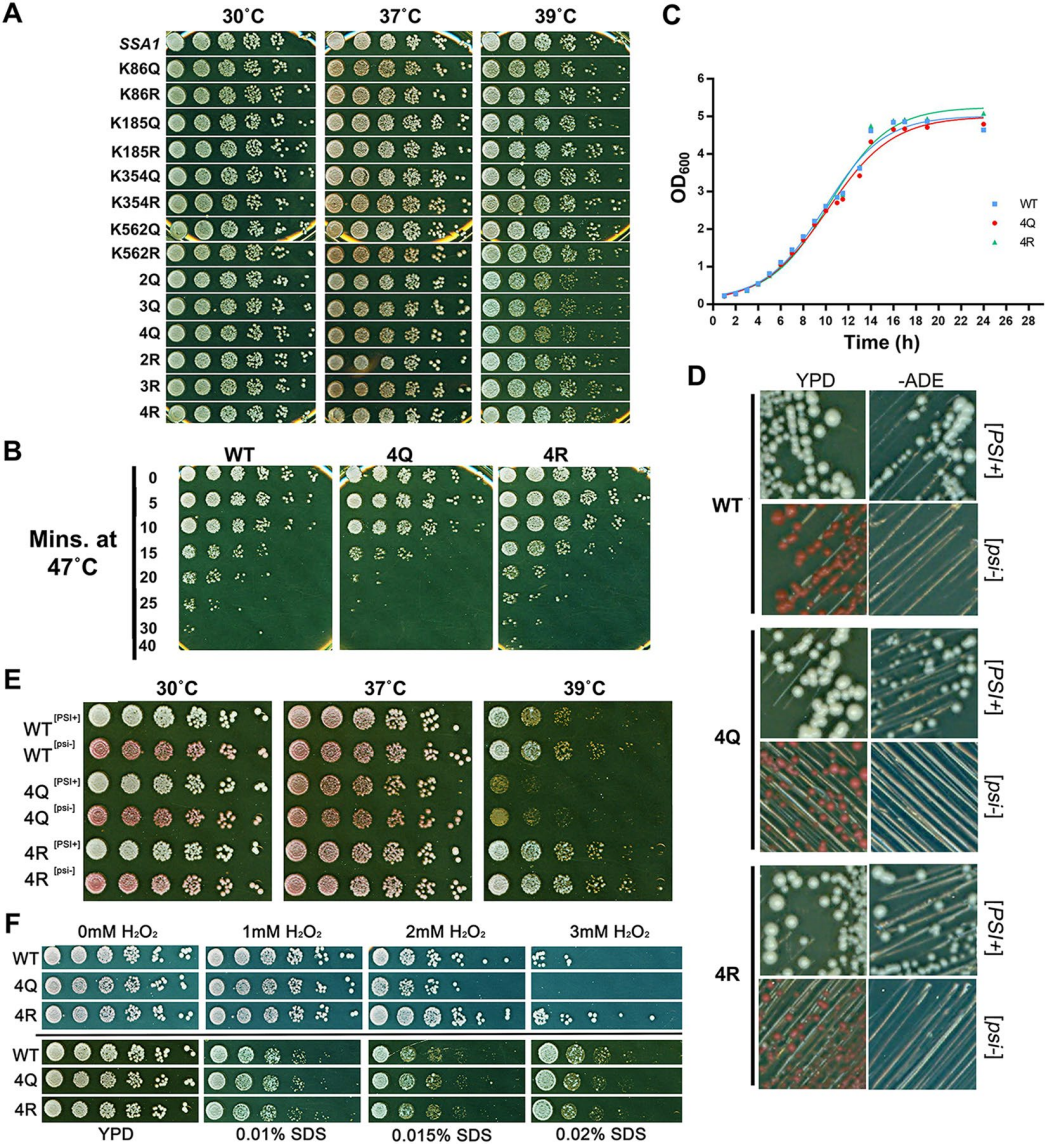
Ssa1 deacetylation alters yeast thermotolerance. (**A**) Yeast expressing acetylation site mutations were grown to exponential phase, serially diluted fivefold and plated onto YPD plates. Plates were photographed after 3 days incubation at the indicated temperatures. (**B**) Assessment of acetylation site mutant recovery after acute heat shock. Fresh cultures were heat shocked at 47 °C for the indicated times and then were serially diluted fivefold and plated onto YPD plates. Plates were photographed after 3 days incubation at 30 °C. (**C**) WT, 4Q and 4R cells were inoculated at an OD_600_ of 0.1 into 96 well format and were shaken in a plate reader at 30 °C. OD_600_ was measured at 1 h intervals. (**D**) Assessment of [*PSI*^+^] propagation. Single colonies of cells expressing WT, 4Q and 4R Ssa1 were streaked on YPD and −ADE plates which were then incubated at RT for 5–7 days. [*psi*^*−*^] cells were red colonies on YPD and unable to grow −ADE plates; [*PSI*^+^] cells were white colonies on YPD and viable on −ADE plates; [*psi*^*−*^] cells were attained by streaking [*PSI*^+^] colonies on YPD plates containing 3 mM GdnHCl and incubating for 2–3 days. The curing of [*psi*^*−*^] cells was repeated at least twice to obtain a stable [*psi*^*−*^] heritage. (**E**) Growth assay of acetylation site mutants for [*PSI*^+^] and [*psi*^*−*^] cells. Yeast were grown and treated as in (**A**). (**F**) Response of acetylation site mutants to stresses that perturb DNA integrity or the yeast cell wall. Cells grown as in (**A**) were five-fold serially diluted and plated onto media containing the indicated stressors.

To assess the phenotypic consequences of Hsp70 acetylation, point mutations were introduced at K86, K185, K354 and K562 to create acetylation-deficient (lysine to arginine, K → R) and acetylation-mimetic (lysine to glutamine, K → Q) mutants. These mutants were expressed in G402 yeast as the sole Ssa protein in the cell. Mutation of any combination of the 4 lysines to R or Q did not affect the ability of Ssa1 to provide essential cell viability (Fig. 2A). A good indicator of *in vivo* chaperone functionality is the ability of yeast to tolerate exposure to heat stress. We examined whether single or multiple (de)acetylation mutants affected ability to grow at 39 °C. None of the individual K → R or K → Q mutation showed temperature sensitivity. However, combining K → Q mutants caused temperature sensitivity at 39 °C with the greatest effect observed for K86Q/K185Q/K354Q/K562Q (4Q) mutants (Fig. 2A). No temperature sensitive phenotypes were observed when combining K → R mutations (Fig. 2A).

We directly analyzed the thermotolerance of the acetyl-mutant strains by observing recovery after exposure to an acute stress of 47 °C. The 4Q mutant was less thermotolerant compared to WT and the 4R strain, especially apparent after 20 mins exposure (Fig. 2B). We also examined growth rates of WT, 4Q and 4R cells in liquid media and detected no significant impact in cell growth at 30 °C (Fig. 2C). To eliminate the possibility of strain specific effects, 4Q or 4R mutants were expressed in an alternative *ssa1-4*∆ yeast strain^25^. In this background, the 4Q mutant also displayed a lack of thermotolerance (Fig. S1).

Chaperones play a critical role in protein folding and it is well established that they regulate the folding and propagation of prions^26^. To determine whether Ssa1 acetylation affects [*PSI*^+^] prion propagation we analyzed the red/white colony pattern from mutant cells, a well-characterized prion marker^27^. 4Q and 4R cells behaved similarly, maintaining the [*PSI*^+^] prion (observed as white colonies and ability to grow on media lacking adenine) (Fig. 2D). In addition, both 4Q and 4R cells could be cured by 3 mM GdnHCl (prion cured [*psi*^−^] cells are red and do not grow on media lacking adenine) (Fig. 2D). Previous reports have shown the [*PSI*^+^] prion can induce read-through of cells carrying nonsense mutations^28^ and alter genome-wide translation^29^. The 4Q mutant, whether [*PSI*^+^] or [*psi*^*−*^], displayed a more pronounced temperature sensitivity at 39 °C (Fig. 2E).

Molecular chaperones contribute to both the DNA damage response (DDR) and cell wall integrity pathways in yeast^30–38^. To investigate whether the acetylation of Ssa1 alters activity of either of these pathways, we tested growth of mutants in the presence of the cell wall perturbing agent SDS and the oxidative stress agent H_2_O_2_. Cells expressing the acetylation-mimetic 4Q variant as the only source of Ssa were sensitive to H_2_O_2_, but not to SDS (Fig. 2F). Interestingly, the 4R variant was clearly more resistant to both H_2_O_2_ stress and heat shock (Fig. 2B,F) compared to WT, which supports the notion that deacetylation plays a role in fine-tuning Hsp70 function in response to a variety of stresses.

### Ssa1 acetylation alters regulation of the heat shock response

**Figure 3:**
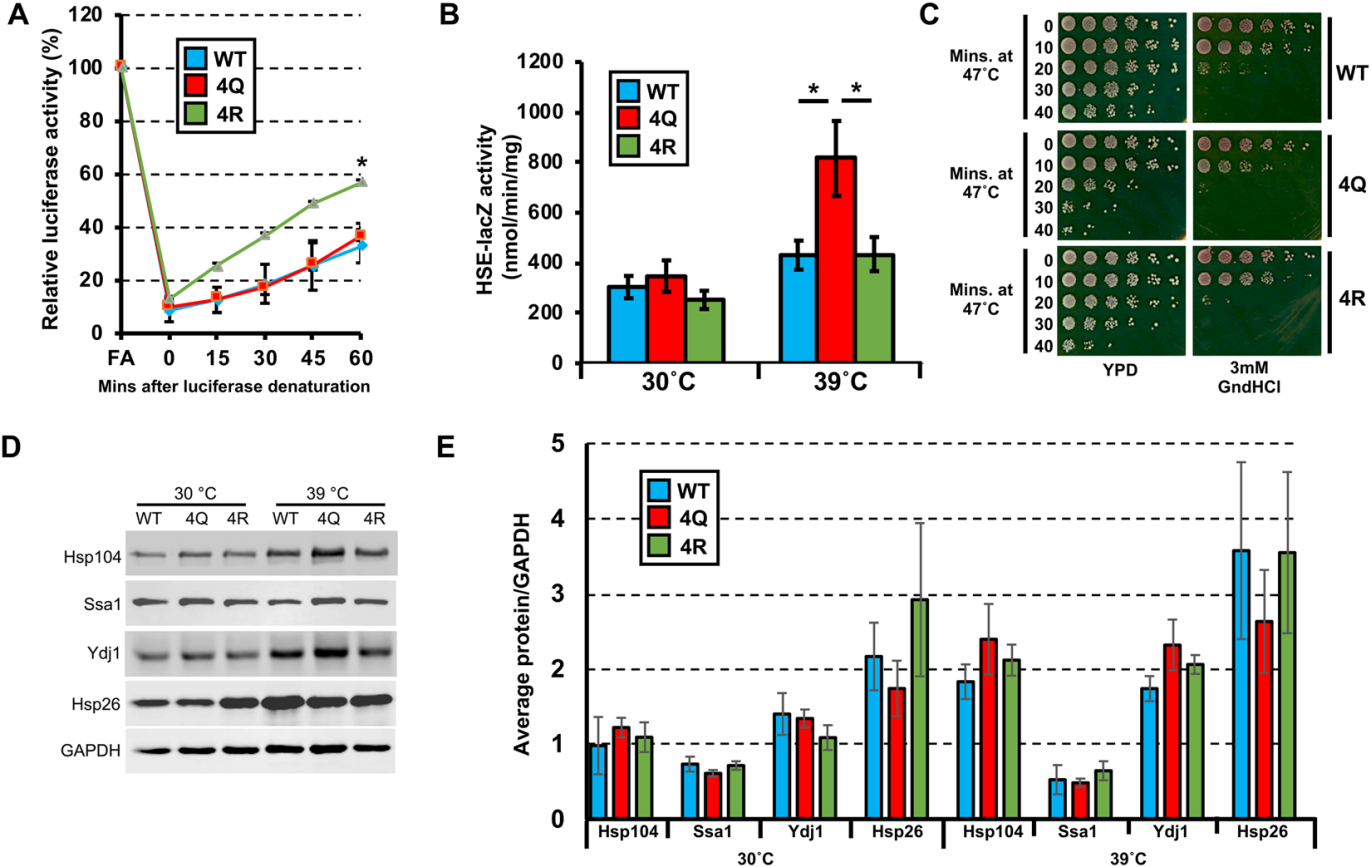
Effects of Ssa1 deacetylation on *in vivo function*. (**A**) Ability of Ssa1 (WT, 4Q and 4R) to refold Luciferase over a 60 min time course. Activity relative to fully active luciferase was calculated and data shown are the average and SD of three independent experiments. (**B**) Effects of acetylation of Ssa1 on HSF1 activation. A plasmid containing the HSE-lacZ reporter gene was transformed into G402 cells. Cells were cultured at 30 °C or heat shock at 39 °C for 2 h. The activation of Hsf1 was calculated by measuring the expression of β-galactosidase under Heat Shock Element (HSE) control. Data are the average and SD of three independent experiments. *Represents p < 0.05. (**C**) Acquired thermotolerance assay of acetylation site mutants. Fresh cultures were pre-treated at 39 °C for 1 h, then cells were heat shocked at 47 °C for the indicated times and then plated on media either containing or lacking 3 mM Gdn-HCl (an inhibitor of Hsp104). (**D**) Steady state levels of major co-chaperone proteins in acetylation site mutants. WT, 4Q and 4R cells were grown to exponential phase and were either incubated at 30 °C or 39 °C for 2 hours. Cell extracts were obtained, resolved on SDS-PAGE gels and analyzed by immunoblotting with anti-Hsp104, Ssa1, Ydj1 and Hsp26 antibodies. GAPDH was used as a loading control. (**E**) Quantitation of major co-chaperone in acetylation site mutants. Data shown are the average and SD of three independent experiments.

Ssa1 plays a major role in both the folding of newly synthesized proteins and the refolding of denatured proteins. To assess the influence of Ssa1 acetylation on Ssa1 protein refolding, we utilized a well-established *in vivo* luciferase refolding assay. Although the 4Q variant maintained similar refolding activity as that of WT, the 4R mutant possessed significantly enhanced refolding activity (Fig. 3A). Hsp70 regulates the heat shock response by binding the heat shock transcription factor (Hsf1), keeping it in a monomeric inactive state in yeast and mammalian cells^2^. To investigate whether Hsp70 acetylation influences Hsf1 activity, we measured the ability of WT, 4Q and 4R cells to activate a well-established *HSE*-*lac*Z reporter^39^ in WT, 4Q and 4R cells at 30 °C and 39 °C. While all of the strains displayed similar basal Hsf1 activity at 30 °C, 4Q produced an elevated heat shock response at 39 °C (Fig. 3B).

As described in our recent work, a C-terminal truncation of Ssa1 ΔGGAP displays a temperature sensitive (*ts*) phenotype related Hsp104 redundancy, which we consider a compensatory mechanism to adapt to heat shock^34^. To clarify whether the temperature sensitivity of the 4Q mutant is connected to Hsp104 function, we assessed the ability of WT, 4Q and 4R cells to grow on media containing 3 mM Gdn-HCl (an inhibitor of Hsp104^40^). Although the 4Q mutant is thermosensitive compared to WT and 4R mutants, addition of Gdn-HCl almost equalized growth of all cells (Fig. 3C). Taken together, these results suggest interplay between Hsp104 function and Ssa1 acetylation in acquisition of thermotolerance.

Hsf1 activity directly drives the transcription of HSP genes such as Hsp104, Ydj1 and Hsp26^41,42^. We considered the possibility that the phenotypes observed in Ssa1 acetylation mutants may be a result of altered chaperone/co-chaperone expression. Analysis of Hsp104, Ssa1, Ydj1 and Hsp26 from unstressed and heat shocked cells determined that Ssa1 had not been destabilized by the 4Q or 4R mutations (Fig. 3D,E). As predicted by the results in Fig. 3B, the Hsp104 and Ydj1 co-chaperones were upregulated in 4Q under heat shock, while 4R displayed increased Hsp26 under untreated conditions (Fig. 3D,E).

### Acetylation alters the interactome of Ssa1

**Figure 4:**
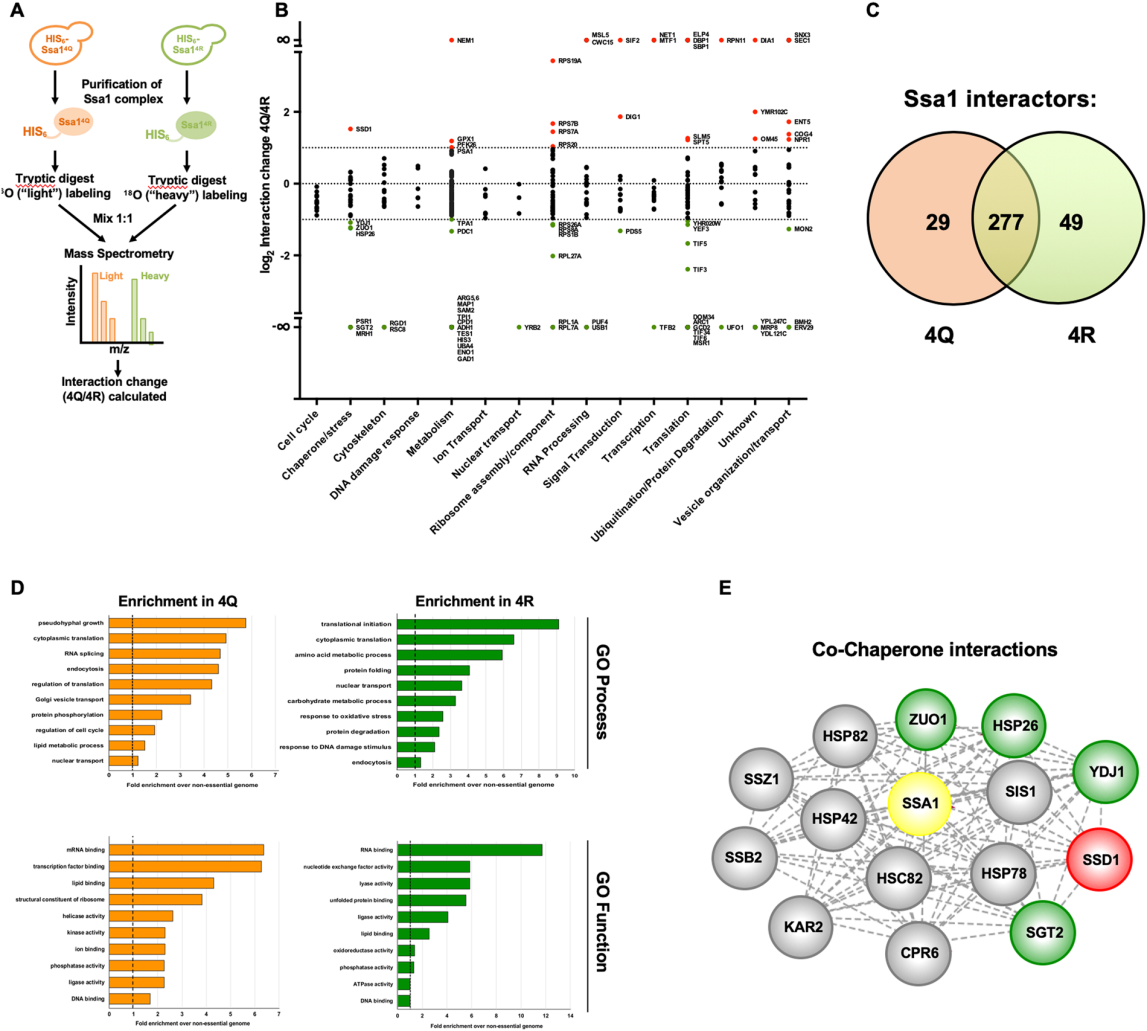
Acetylation alters the interactome of Ssa1. (**A**) Scheme for proteomic analysis. Cells expressing 4Q or 4R mutant His_6_-Ssa1 were grown to exponential phase, whereupon Ssa1 complexes were affinity purified and digested with trypsin. Peptides from 4Q interactors were isotopically labeled with ^18^O, mixed 1:1 with 4R interactor peptides, and analyzed by quantitative LC-MS/MS. (**B**) Functional classification of the Ssa1 interactome. Ssa1 interactors were categorized by cellular function using Gene Ontology (GO) Slim analysis and plotted against relative affinity for 4Q vs 4R Ssa1. The dotted lines represent a ratio of two-fold. Interactors are colored by relative selectivity to 4Q or 4R Ssa1 as follows: red (significant selectivity for 4Q), green (significant selectivity for 4R), black (equal binding to 4Q and 4R). (**C**) Venn diagram of Ssa1 interactors observed in 4Q and 4R interactomes after applying statistical filters. (**D**) Gene Ontology (GO) term analysis of 4Q and 4R interactomes. Interactors were categorized by cellular function using GO Slim analysis and relative enrichment compared to occurrence in the non-essential genome was calculated. The top 10 enriched cellular processes and function are shown for both 4Q and 4R interactomes. (**E**) Analysis of chaperone/co-chaperone interactions of 4Q and 4R Ssa1. A network of the 15 co-chaperones and chaperones that were detected by mass spectrometry as interactors of 4Q and 4R Ssa1 was generated using Cytoscape. The nodes were colored based on relative binding preference for 4Q and 4R as follows: red (selectivity for 4Q), green (selectivity for 4R) and gray (no preference). Raw mass spectrometry data are available via ProteomeXchange with identifier PXD015185.

Co-chaperones bind to Hsp70 primarily at the N-terminus, close to the region responsible for ATP binding and hydrolysis^3,43,44^. In contrast, it is thought that the majority of client binding and refolding takes place at the C-terminal substrate binding domain^3^. The four acetylation sites K86, K185, K354 and K562Q are distributed through N and C terminal domains, making it likely that acetylation of these sites causes both structural changes and alteration of Ssa1 interactions. We therefore employed global proteomics to assess interactome changes of Ssa1 in response to acetylation. We purified Ssa1 complexes from 4Q and 4R cells grown at 30 °C via HIS-dynabeads and compared them quantitatively via isotope-coded tandem mass spectrometry (LC-MS/MS, Fig. 4A). The list of Ssa1 4Q and 4R protein interactors is provided in Table S1 and the raw data are available via ProteomeXchange with identifier PXD015185. The average log ratio-change in interaction (4Q vs. 4R) for each quantitated interacting protein was calculated and change in interaction greater than two-fold up or down (>1, <−1) was considered significant. We sorted interactomes by non-redundant GO terms and plotted their average change in interaction upon acetylation (Fig. 4B). Consistent with the idea that PTMs on chaperones fine-tune their function in subtle ways, out of the 355 total protein interactions detected, 277 (78%) bound equally to 4Q and 4R Ssa1, 29 (8%) displayed preferential binding for 4Q Ssa1 and 49 (14%) were selective for the 4R version (Fig. 4B,C). Additionally, we identified 14 (4%) unique 4Q interactions and 35 (10%) unique 4R interactions (Fig. 4B, Table S1). Gene ontology (GO) analysis of proteins that preferentially bound 4Q or 4R forms revealed pathway selectivity. Proteins involved in pseudohyphal growth, RNA splicing, vesicle transport, protein phosphorylation, cell cycle regulation and lipid metabolism were uniquely enriched in 4Q complexes (Fig. 4D). In contrast, proteins involved in amino acid metabolism, carbohydrate metabolism, response to DNA damage and protein degradation were enriched in 4R complexes (Fig. 4D). The majority of Hsp70-client interactions are mediated through interactions with co-chaperones proteins. We noted that several major co-chaperones including Ydj1, Zuo1, Sgt2 and Hsp26 preferentially bound 4R Ssa1 (Fig. 4B,E).

### Acetylation of Ssa1 modulates respiratory capacity during heat shock

**Figure 5:**
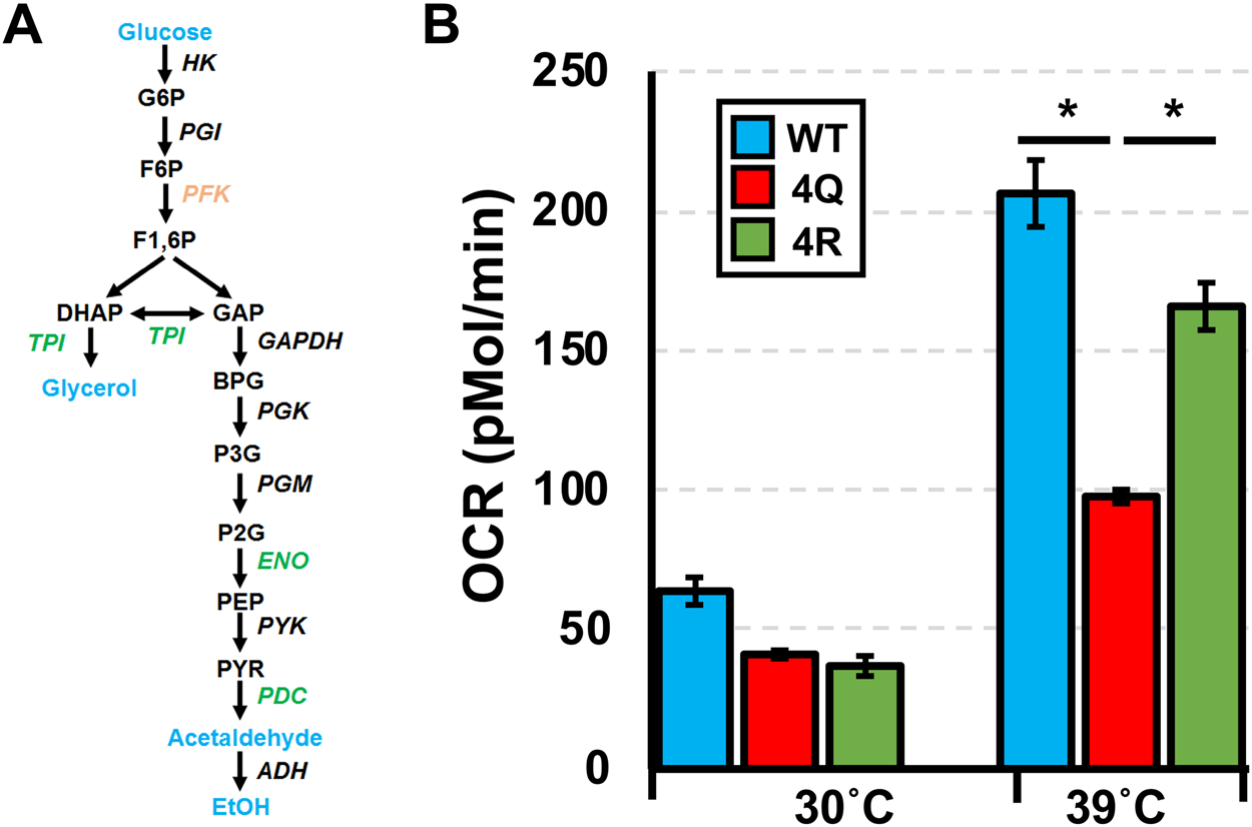
Acetylation of Ssa1 alters metabolism. (**A**) Glycolysis pathway highlighting protein interactors of Ssa1 altered by acetylation (4Q, orange) or deacetylation (4R, in green) from the interactome. (**B**) Assay of oxygen consumption rate. The oxygen consumption rate (OCR) was measured by a seahorse FXe96. Data shown are the average and SD of six independent replicates. *Represents p < 0.01.

The cellular response to stress is closely linked to metabolism. Mitochondria influence the regulation of the stress response and increased temperature enhances the cellular consumption of oxygen^45^. Respiration deficient mutants are less resistant to oxidative stress (H_2_O_2_) and heat shock^46^. Notably, metabolic processes displayed the most significant unique interaction changes upon Hsp70 deacetylation (Fig. 4B) and enrichment analysis suggested a difference in carbohydrate metabolism (Fig. 4D). 4R Ssa1 preferentially interacts with key enzymes in glycolysis (Fig. 5A). Therefore, we investigated the oxygen respiration rate under heat shock conditions. The basal oxygen respiration rates (OCR) were similar in all samples at normal temperature, while WT and 4R showed 3-fold enhanced basal respiration compared to 4Q at elevated temperatures (Fig. 5B). Although 4Q cells are temperature-sensitive, 2 h heat shock at 39 °C did not cause cells death (Fig. S2). The OCR decrease of 4Q would thus appear to be a direct or indirect result of a change in the Ssa1 interactome due to deacetylation.

## Discussion

Our data show that high temperature exposure triggers rapid deacetylation of Ssa1 at K86, K185, K354 and K562, suggesting a requirement for Ssa1 deacetylation in the heat shock response (HSR). This indeed appears to be the case as mutation of these sites to mimic constitutive acetylation (4Q) results in increased thermosensitivity and correspondingly, 4R cells are relatively thermoresistant. These phenotypes appears to be linked to altered regulation of Hsf1-mediated transcription as seen by both HSE-reporter readout and compromised co-chaperone expression. While the increase in activation of the heat shock response in the 4Q mutant at first appears counterintuitive, we believe it is reflective of functional changes in Ssa1 that result in a reduction in the ability to deal with the consequences of increased temperature, and hence result in an over-stimulation/compensation of the heat shock response.

We demonstrate here that deacetylation of Ssa1 alters core functional activities of the protein, such as aiding the re-folding of denatured proteins. It may be that Ssa1-Hsf1 interaction is modulated by Ssa1 acetylation. Alternatively, Ssa1-client interaction kinetics may change, both at the level of client interaction and folding capability. The effects of Ssa1 acetylation appear to be fairly specific; the 4Q and 4R mutants show unchanged resistance to both cell wall damaging and oxidative stress agents, resistance of which is dependent on Ssa1 function. Post-translational modification of major chaperones that include Hsp70 and Hsp90 result in wide-scale remodeling of client and co-chaperone interactions^15,47–52^. K86, K185 and K354 all reside in the N-terminal domain of Ssa1, an area known to be critical for both co-chaperone binding and ATP binding and hydrolysis^3,43,44^. Mimicking acetylation at these four residues caused a significant shift in the interactome of Ssa1, causing a loss of interaction with key co-chaperones such as Ydj1, Zuo1, Sgt2 and Hsp26. Loss of these interactions (but not Ssa1-Sis1) upon Ssa1 acetylation is remarkably similar to the interactome change seen upon T36 phosphorylation^15^ and may indicate a mechanism to regulate cell growth under different cellular stresses. K562 resides in the client binding domain and may explain the large loss of client protein association upon Ssa1 acetylation, especially those associated with metabolism and translation. Significant changes in the Hsp70 interactome with proteins involved in glycolysis results in clear changes to respiration *in vivo*, which may also contribute to phenotypic differences between 4Q and 4R. While not detected in our mass spectrometry experiment FBPase is also a client of Ssa1, an enzyme that converts fructose 1, 6-biphosphate to fructose 6-phosphate^36,53–55^. This reaction is the inverse of the reaction catalyzed by PFK1, detected as an Ssa1 interactor in this study. It may be that Ssa1 modification alters interaction FBPase and PFK1, altering the relative activities and controlling the directional flow through the pathway in response to differential energy requirements.

It should be noted that while there is substantial differences between the 4Q and 4R interactomes, 78% of Ssa1 interactions remained unchanged. This data along with the relatively subtle phenotypes observed suggests that deacetylation upon heat shock is fine-tuning Ssa1 function while leaving essential functions intact.

Heat stress causes protein denaturation and aggregation and cells must respond rapidly to maintain viability. A well-established model of the HSR involves competition of binding between Hsf1, Ssa1 and denatured protein clients. As more proteins become unfolded, Ssa1 is titrated away from Hsf1, allowing full activation of Hsf1-mediated genes, including a variety of inducible chaperones. While this response has been well characterized in several organisms, this response is limited by the speed of cellular transcription and translation, working over longer time frames. Recent studies have demonstrated that cells can respond much more rapidly to acute heat shock. For example, experiments heat shocking yeast at 46 °C for 2–8 minutes show mass aggregation and redistribution of proteins into stress granules^56^. It is interesting to note that these aggregates are not misfolded and are not targeted for degradation. These aggregates are resolved post-shock which suggests they play a protective role in the HSR. It is intriguing to speculate that Ssa1 deacetylation may be linked to this process and may be even a key trigger in the formation/resolving of these reversible aggregates.

Combinations of the PTMs present in the chaperone code present a variety of challenges in terms of understanding PTM stoichiometry and the diversity of modifications (proteoforms) present. While our mass spectrometry experiments have uncovered altered acetylation on Ssa1, we cannot accurately quantify the proportion of Ssa1 molecules acetylated in unstressed conditions or whether the identified PTMs are located on the same Ssa1 molecule. The most likely scenario is that under non-stressed conditions Ssa1 exists in a population of differentially acetylated states, rather than defined all or nothing status.

Yeast express four cytosolic Ssa proteins, two constitutive (Ssa1 and Ssa2) and two heat-inducible forms (Ssa3 and Ssa4). These isoforms appear to have high functional overlap, especially as yeast can maintain viability upon loss of any three Ssa isoforms^24^. Recent evidence suggests that these Hsp70 isoforms are not fully redundant and have specialized roles dictated though unique clientomes^24,57,58^. Although 3 of 4 lysines are conserved in the Ssa proteins, the heat inducible Ssa3 and Ssa4 contain an alanine at position 562 instead of lysine, and thus cannot undergo acetylation at this site. This raises the intriguing possibility that for the constitutively expressed Ssa1 to function as well as Ssa3 or Ssa4 under stress conditions, deacetylation at K562 needs to occur. For the first time, we may be observing chaperone code modifications that switches chaperone specificity from one isoform to another. Future high-resolution studies on the Ssa interactomes under a variety of stresses should hopefully confirm this theory.

## Materials and Methods

### Plasmids and yeast strains

The *SSA1* gene and its mutations were constructed in the pC210 plasmid^59^ using a site-directed mutagenesis kit (Agilent) and primers indicated in Table S1. The plasmids were transferred into the G402 background (*MATa ade2-1 SUQ5 kar1-1 his3 leu2 lys2 trp1 ura3 ssa1:: KanMX*, *ssa2::HIS3*, *ssa3::TRP1*, *ssa4::URA3-1f/pRDW10*)^35^. Colonies were then streaked onto plates containing 5-fluoroorotic acid in order to select against the resident *URA3* plasmid containing the wide-type *SSA1*.

### Yeast growth assays

Yeast strains were cultured in 5 ml YPD or SC with the appropriate selection media at 30 °C overnight. The following morning, yeast cultures were diluted in 6 ml of the appropriate selection media at an OD_600_ of 0.2 and cultured again until OD_600_ 0.5 was reached. A 1/5 serial dilution was performed in a 96-well plate and then replicated onto YPD or appropriate media. Plates were incubated at 30 °C or elevated temperatures (37 °C and 39 °C) for 2 days. For thermotolerance assays, cells (OD_600_ of 0.5) were cultured at 47 °C heat shock and aliquots of cells were taken at indicated time points, followed by a 1/5 serial dilution and plating on YPD and incubation for 2 days. For acquired thermotolerance assays, cells (OD_600_ of 0.5) were pre-heated at 39 °C for 2 h and then cultured at 47 °C (or 50 °C for MH272) heat shock at an interval of every 10 min for a duration of 40 min, followed by a 1/5 serial dilution and plating on YPD and incubation for 2 days. For growth assays using oxidative stress agents and cell wall damaging agents, cells (OD_600_ of 0.5) were spotted onto SC-H_2_O_2_ (0 mM, 1 mM, 2 mM and 3 mM) or YPD containing SDS (0, 0.01%, 0.015% and 0.02%, w/v) respectively.

### Protein extraction from yeast

Yeast strains were cultured overnight in 5 ml YPD or selective media at 30 °C. The following morning, cells were diluted in 25 ml of fresh media to an OD_600_ of 0.2 and incubated until an OD_600_ of 0.6–0.8 was reached. Cells were harvested by centrifugation at 4 °C (5 min, 2500 rpm) and pellets were washed with distilled water. Pellets were resuspended in yeast cell lysis reagent (Sigma) with 10 mM DTT and protease inhibitor cocktail (Sigma). Glass beads of 0.5 mm diameter were added to aid cell lysis using a mini-beater (Biospec products). Supernatants were transferred to pre-chilled 1.5 ml microfuge tubes and centrifuged (10 min, 13000 rpm) to remove any cell fragments.

### Trypsin digestion of samples from SDS-PAGE gel plugs

IP eluates were loaded onto a 12% MOPS buffered SDS-PAGE gel (Invitrogen) and run for 15 min at 200 V resulting in a ~3 cm “gel plug”. The gel was stained with 25 mL Imperial Stain (Pierce) at room temperature, and destained overnight in dH_2_O at 4 °C. The gel plugs for each sample to be analyzed were excised by sterile razor blade, one ~3 cm section per sample, and chopped into ~1 mm^3^ pieces. Each section was washed in dH_2_O and destained using 100 mM NH_4_HCO_3_ pH 7.5 in 50% acetonitrile. A reduction step was performed by addition of 100 μl 50 mM NH_4_HCO_3_ pH 7.5 and 10 μl of 200 mM tris(2-carboxyethyl)phosphine HCl at 37 °C for 30 min. The proteins were alkylated by addition of 100 μl of 50 mM iodoacetamide prepared fresh in 50 mM NH_4_HCO_3_ pH 7.5 buffer and allowed to react in the dark at 20 °C for 30 min. Gel sections were washed in water, then acetonitrile, and vacuum dried. Trypsin digestion was carried out overnight at 37 °C with 1:50–1:100 enzyme–protein ratio of sequencing grade-modified trypsin (Promega) in 50 mM NH_4_HCO_3_ pH 7.5, and 20 mM CaCl_2_. Peptides were extracted with 5% formic acid and vacuum dried.

### HPLC for mass spectrometry

All samples were re-suspended in Burdick & Jackson HPLC-grade water containing 0.2% formic acid (Fluka), 0.1% TFA (Pierce), and 0.002% Zwittergent 3–16 (Calbiochem), a sulfobetaine detergent that contributes the following distinct peaks at the end of chromatograms: MH+ at 392, and in-source dimer [2 M + H+] at 783, and some minor impurities of Zwittergent 3–12 seen as MH+ at 336. The peptide samples were loaded onto a 0.25 μl C8 OptiPak trapping cartridge custom-packed with Michrom Magic (Optimize Technologies) C8, washed, then switched in-line with a 20 cm by 75 μm C18 packed spray tip nano column packed with Michrom Magic C18AQ, for a two-step gradient. Mobile phase A was water/acetonitrile/formic acid (98/2/0.2) and mobile phase B was acetonitrile/isopropanol/water/formic acid (80/10/10/0.2). Using a flow rate of 350 μl/min, a 90 min, 2-step LC gradient was run from 5% B to 50% B in 60 min, followed by 50%–95% B over the next 10 min, hold 10 min at 95% B, back to starting conditions and re-equilibrated.

### LC–MS/MS and statistical analysis

The samples were analyzed via electrospray tandem mass spectrometry (LC–MS/MS) on a Thermo Q-Exactive Orbitrap mass spectrometer, using a 70,000 RP survey scan in profile mode, m/z 360–2000 Da, with lock masses, followed by 10 MSMS HCD fragmentation scans at 17,500 resolution on doubly and triply charged precursors. Single charged ions were excluded, and ions selected for MS/MS were placed on an exclusion list for 60 s.

All LC-MS/MS *.raw Data files were analyzed with MaxQuant version 1.5.2.8, searching against the SPROT *Saccharomyces cerevisiae* database downloaded 1/9/2018 and searched using the following criteria: LFQ quantification with a min of 1 high confidence peptide. Trypsin was selected as the protease with max miss cleavage set to 2. Carbamiodomethyl (C) was selected as a fixed modification. Variable modifications were set to Deamidation (NQ), Oxidization (M), Formylation (n-term), and Phosphorylation (STY). Orbitrap mass spectrometer was selected using a MS error of 20 ppm and a MS/MS error of 0.5 Da. A 1% FDR cutoff was selected for peptide, protein, and site identifications. LFQ Intensities were reported based on the MS level peak areas determined by MaxQuant. Proteins were removed from this results file if they were flagged by MaxQuant as “Contaminants”, “Reverse” or “Only identified by site”. Three complete biological replicates were performed. The abundance data from each biological replicate were normalized to the ratio of Ssa1. LFQ Peak intensities were analyzed in each run to determine protein hits that fell into the category of either Unstressed only hits or Stressed only hits and retained if they confirmed to this state across all 3 runs. LFQ Significance cutoffs are Significantly Up >1.2 ratio (Log_2_ 0.26) and Significantly Down <0.8 ratio (Log_2_ −0.32). Any hits that were not observed in at least 2 replicates each were labeled ‘no quant’ (a normalized ratio was still calculated but not included in final data set analysis).

### Luciferase refolding assay

Luciferase assay was carried out as previously described^35^. Strains were transformed with plasmid pDCM90, a URA3-based low-copy plasmid containing a gene for expression of a thermolabile bacterial luciferase LuxAB [35]. Transformed strains containing pDCM90 were cultured in 5 ml selective medium without uracil at 30 °C overnight. The following day, cultures were diluted to OD_600_ of 0.2 into the same medium and incubated at 37 °C shaking for 30 min to induce expression of heat shock proteins. After culturing, the cellular luciferase activity of each strain was measured by immediately adding 10 µl of decanal (Sigma) to 200 µl of culture in a FB12 Luminometer (Berthold Detection Systems) as a reading for 100% activity. Cells were then transferred to a 45 °C shaking incubator for 1 h. During this 1 h heat shock, cyclohexamide (Sigma) was added after 50 min at a concentration of 10 µg/ml. After thermal denaturation at 45 °C, cellular luciferase activity was measured as start point (0 min). Cultures were shifted to 25 °C at an interval of every 15 min for duration of 1 h. Luciferase recovery was calculated as a percentage of the 100% activity.

### β-Galactosidase assay

β-Galactosidase assay was carried out as previously described^60^. The chemical reagents used in this assay were purchased from Sigma. Before beginning the assay, a plasmid with *lacZ* under HSE regulation was transformed into the appropriate strains. This is because *HSF1* binds with HSE and activates gene expression. The β-Galactosidase assay was employed to indirectly calculate activity of Hsf1 *in vivo*. The quantity of cell extract used was 20 μg in this assay. After protein extraction, the assay was performed using a 28 °C water bath for approximately 30 min or until a yellow color develops. The finally reaction mixture was measured at OD_420_ in triplicate. The activity of LacZ is expressed by the following formula:

$${\rm{Activity}}\,{\rm{of}}\,{\rm{LacZ}}=\frac{OD420\times 1.7}{0.0045\times P\times T}$$

where OD_420_ is the optical density of the product o-nitrophenol at 420 nm; a correction factor of 1.7 is applied to the reaction volume; the factor 0.0045 is the OD of a 1 nmol/ml solution of the product; P is the amount of protein lysate in mg; and T is reaction time in minutes. The specific activity of LacZ is expressed as nmoles/min/mg of lysate protein.

### Basal respiration assay

Basal respiration was evaluated by the oxygen consumption rate (OCR) measurements carried out as previously described with minor modifications^61^. Yeast cells were cultured overnight at 30 °C in SC medium. Cells were diluted into fresh medium and cultured until they reached an OD_600_ of 0.5. The cell density was adjusted and a 180 µl medium containing 2 × 10^5^ cells was seeded in a XF 96-well microplates (Seahorse Bioscience). Following 1 h incubation at 30 °C or 39 °C, OCR was measured according to the manufacturer’s manual on a Seahorse XFe96 Extracellular Flux Analyzer.

## Supplementary information

Figures S1-3

Table T1

## Data Availability

The mass spectrometry proteomics data have been deposited to the ProteomeXchange Consortium via the PRIDE (https://www.ebi.ac.uk/pride/archive/) partner repository with the dataset identifier PXD015185.
