# Supplementary material for: Rapid deacetylation of yeast Hsp70 mediates the cellular response to heat stress
Source: Sci Rep. 2019 Nov 7;9:16260. doi: 10.1038/s41598-019-52545-3 (PMC6838335; doi:10.1038/s41598-019-52545-3)
Supplement: Supplementary file 1 — Figures S1-3 [file 41598_2019_52545_MOESM1_ESM.pdf]

Supplementary information for:

Rapid deacetylation of yeast Hsp70 mediates the cellular response to heat stress

Linan Xu[1], Nitika[2], Naushaba Hasin[3], Daragh D. Cuskelly[1], Donald Wolfgeher[4], Sean Doyle[1], Paul Moynagh[1], Sarah Perrett[5,6], Gary W. Jones[1,7] *, Andrew W. Truman[2]*

[1]Department of Biology, Maynooth University, Maynooth, Co. Kildare, Ireland.
[2]Department of Biological Sciences, University of North Carolina at Charlotte, Charlotte, USA.
[3]Institute for Genome Sciences, University of Maryland Baltimore, Baltimore, USA
[4]Department of Molecular Genetics and Cell Biology, The University of Chicago, Chicago, USA
[5]National Laboratory of Biomacromolecules, CAS Center for Excellence in Biomacromolecules, Institute of Biophysics, Chinese Academy of Sciences, Beijing 100101, China.
[6]University of the Chinese Academy of Sciences, 19A Yuquan Road, Shijingshan District, Beijing 100049, China.
[7]Centre for Biomedical Science Research, School of Clinical and Applied, Leeds Beckett University, Portland Building, City Campus, Leeds LS1 3HE, United Kingdom.

*Corresponding authors
E-mail: gary.jones@leedsbeckett.ac.uk (GJ) or A.Truman@uncc.edu (AT)

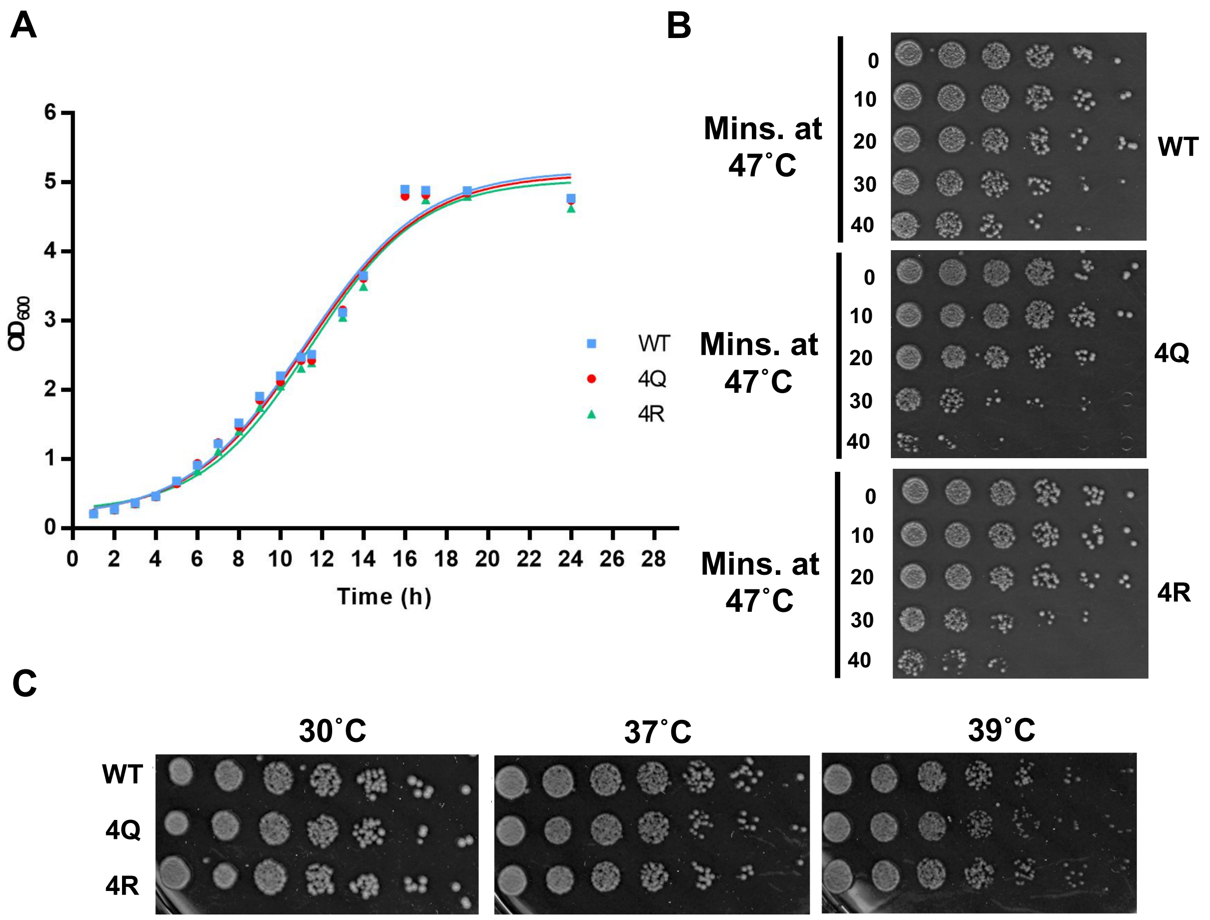

Fig. S1. Growth evaluation of MH272 strains. (A) Growth curves of MH272 strains at 30˚C in YPD. (B) Growth assay of the (de)acetylated mutations in Ssa1 at elevated temperatures. (C) Acquired thermotolerance assay of (de)acetylation mutations of Ssa1 at extreme temperature. Fresh cultures were pre-treated at 39˚C for 1 h, then cells were heat shocked in a 50˚C incubator for a time course (T0-T4 means 0, 10, 20, 30 and 40 min, respectively).

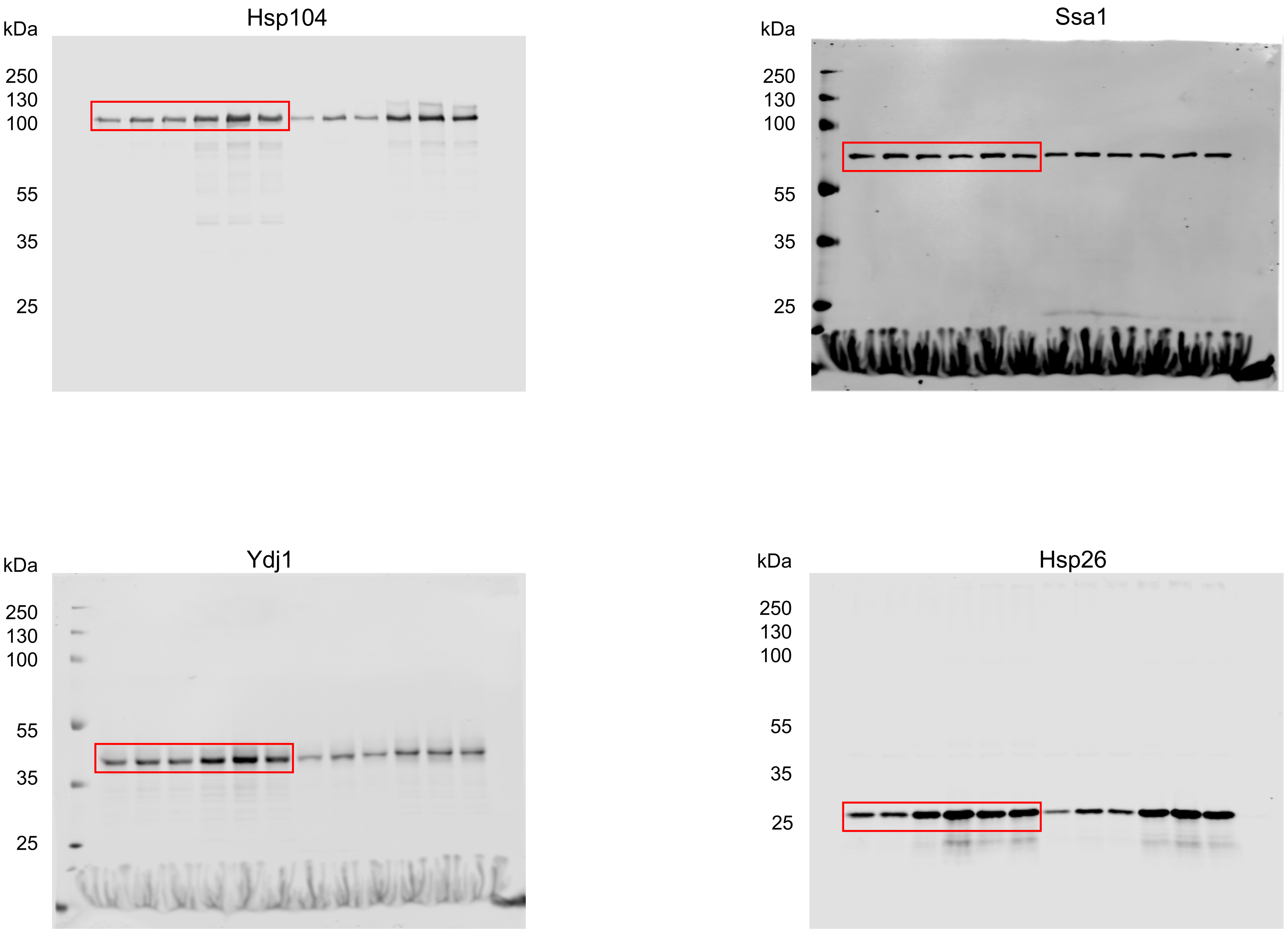

Fig. S2. Original blots for data shown in Fig. 3D. Regions used in Fig. 3D are highlighted in red. MW are labelled along with primary antibody used.

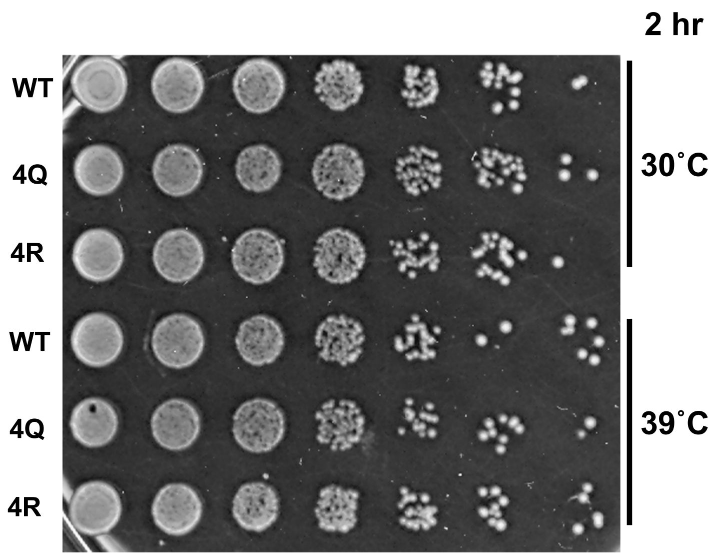

Fig. S3. Viability G402 strains post-heat shock. Yeast expressing acetylation site mutations were grown to exponential phase, and then fresh cultures were heat shocked at 39˚C for 2 h. Cells serially diluted fivefold and plated onto YPD plates. Plates were photographed after 2 days incubation at 30˚C.
